# The Main Causes of Bacterial Colonization in Endotracheal Tubes and Tracheal Secretions in Neonates Admitted to the Neonatal Intensive Care Unit

**Published:** 2017-06

**Authors:** Bita Najafian, Mohammad Torkaman, Ehsan Shahverdi, Reza Noroozian

**Affiliations:** 1 Department of Pediatrics, Baqiyatallah University of Medical Sciences, Tehran, Iran,; 2 Blood Transfusion Research Center, High Institute for Research and Education in Transfusion Medicine, Tehran, Iran,; 3 Blood and Cancer Research Center, MAHAK Pediatric Cancer Treatment and Research Center, Tehran, Iran

**Keywords:** Ventilator-associated pneumonia, Neonatal intensive care unit, Trachea, Culture, Antibiotics

## Abstract

**Background:**

Ventilator-associated pneumonia (VAP) is the second most common nosocomial infection in neonates, admitted to neonatal intensive care units (NICUs). The aim of this study was to identify the main causes of bacterial colonization in endotracheal tubes and tracheal secretions of neonates hospitalized in the NICUs of our university hospitals.

**Materials and Methods:**

This cross sectional study was conducted during 2015–2016. Thirty-nine neonates, who were under mechanical ventilation in the NICUs of Baqiyatallah and Najmiyeh hospitals of Tehran, Iran, were assessed. The patients were selected using the census sampling method. Gestational age, birth weight, duration of mechanical ventilation, length of hospital stay, tracheal discharge culture, endotracheal tube culture, blood culture, and chest radiography were evaluated.

**Results:**

In a total of 39 neonates (50.3% males and 49.7% females) with the mean age of 1.17±1.12 days, bacterial growth was reported in 6 (15.3%) cases. The antibiotic study of tracheal secretion and endotracheal tube cultures showed that 2.6% of patients were resistant to cephalosporins, aminoglycosides, nitrofurantoin, and carbapenem. Moreover, 12.8% were also resistant to fluoroquinolones, besides these antibiotics.

**Conclusion:**

Tracheal secretion culture is a simple and proper approach for the diagnosis of VAP. Besides reducing the costs, this method can play a significant role in decreasing the incidence of infections.

## INTRODUCTION

Ventilator-associated pneumonia (VAP) is defined as nosocomial pneumonia in mechanically ventilated patients, which often occurs within 48 hours after the initiation of mechanical ventilation (1). It is the second most common nosocomial infection in infants and is associated with increased length of hospital stay, leading to high morbidity and mortality among neonatal intensive care unit (NICU) patients, with an estimated incidence of 6–32% (2–5). The origins of VAP are aspiration of secretion, colonization of the airways, use of contaminated instruments, and medications (6).

Generally, understanding the microbiology of VAP is critical for selecting an empirical antibiotic therapy. Gram-negative bacteria are the most commonly isolated causative organisms, while Gram-positive bacteria have become increasingly more common over the past several years (7, 8). The clinical criteria for the diagnosis of VAP have been established by the National Nosocomial Infection Surveillance System (NNIS) and the Center for Disease Control and Prevention (CDC) (9). However, it should be noted that there is no current gold standard for the diagnosis of VAP in neonates (1, 10).

The aim of this study was to identify the main causes of bacterial colonization in endotracheal tubes and tracheal secretions of neonates, hospitalized in the NICUs of our university hospitals.

## MATERIALS AND METHODS

This cross sectional study was conducted during 2015–2016. A total of 39 neonates, who were under mechanical ventilation in the NICUs of Baqiyatallah and Najmiyeh university hospitals (Tehran, Iran), were assessed. The patients were selected using census sampling. Samples of tracheal secretions and endotracheal tubes were collected from the patients, who met the Infectious Diseases Society of America (IDSA) criteria for VAP. According to these criteria, VAP is defined as pneumonia, which develops 48 hours or longer after mechanical ventilation or thereafter following endotracheal intubation. It is characterized by the presence of new or progressive infiltrates, signs of systemic infection (e.g., fever and altered white blood cell count), changes in sputum characteristics, and detection of a causative agent (11).

In this study, the specimen inside the sterilized centrifuge tube, along with 1 cc of sterile physiological saline or normal sterile saline, was centrifuged for 10 minutes at 2000–2500 rpm. In addition, eosin methylene blue and blood agar media were used. The positive samples (>10^5^ bacterial colonies) were transferred to Mueller-Hinton Agar for antibiogram studies. Gestational age, birth weight, duration of mechanical ventilation, length of hospital stay, tracheal discharge culture, endotracheal tube culture, blood culture, and chest radiography were also evaluated. The results were confirmed by a laboratory technologist with 10 years of relevant experience.

All neonates, aged 1 to 28 days with at least 48 hours of mechanical ventilation, were enrolled in this study. The parents completed the informed consent forms for participation in the study. On the other hand, the do-not-resuscitate (DNR) and near-death patients, as well as those with less than 48 hours of mechanical ventilation, were excluded from the study. Patients with cardiovascular diseases, electrolyte imbalance, respiratory distress syndrome, and other underlying pulmonary problems were also excluded.

### Statistical analysis

Data were analyzed using SPSS version 16 for Windows (SPSS Inc. Chicago, IL). Variables with a normal distribution, according to one-sample Kolmogorov–Smirnov test, were compared using independent sample *t* test between the groups and paired sample *t* test within the groups. Chi square test was also used to compare categorical variables in the groups. *P*-value less than 0.05 was considered statistically significant.

### Ethical considerations

This study was approved by the Ethics Committee of Baqiyatallah University of Medical Sciences and Health Services. The parents were asked to sign an informed consent form before completing the questionnaires. All terms of the Declaration of Helsinki were considered, and the patients’ personal information remained anonymous.

## RESULTS

In a total of 39 neonates (50.3% males and 49.7% females), with the mean age of 1.17±1.12 days, 7 (17.9%) cases were born via normal vaginal delivery and 32 (82.1%) cases via cesarean section. In 33 neonates, no organisms were found in the tracheal secretion culture. However, bacterial growth was reported in 6 (15.3%) patients (Table 1); in fact, the endotracheal tube culture was reported positive in 6 patients.

**Table 1.** Demographic Data of understudy patients

	**Total**	**Positive**	**Negative**	**P Value**
**Gender (Male/Female)**	24/15	21/12	3/3	0.528
**Age**	1.17 ± 1.12d			
**Gestational age**	34.15 ± 4.02w	34.27 ± 4.1w	33.5 ± 3.6w	0.672
**Birth weight**	2366.66 ± 844.93g	2394.24 ± 833.3g	2215 ± 715.64g	0.639
**Duration of Mechanical Ventilation**	7.86 ± 7.42d	6.56 ± 5.04d	14.83 ± 13.48d	0.540
**Duration of hospitalization**	18.87 ± 12.79d	16.18 ± 10.01d	33.66 ± 17.10d	0.195
**Type of delivery (NVD/CS)**	7/32	7/26	0/6	0.213
**Tracheal discharge culture**			33 (84.6%)	
	Enterobacter	2 (5.1%)		
	Acinetobacter	2 (5.1%)		
	Pseudomonas	2 (5.1%)		
**Endotracheal tube culture**			33 (84.6%)	
	Enterobacter	3 (7.71%)		
	Acinetobacter	2 (5.1%)		
	Pseudomonas	1 (2.6%)		
**Blood culture**			36(92.3%)	
	Streptococcus	1(2.6%)		
	Staphylococcus Saprophyticus	1(2.6%)		
	Gram-negative bacilli	1(2.6%)		

The antibiotic study of tracheal secretion and endotracheal tube cultures showed that 2.6% of patients from each group were resistant to cephalosporin, aminoglycosides, nitrofurantoin, and carbapenem, respectively. Moreover, 12.8% of patients were resistant to fluoroquinolones, besides these antibiotics. The blood culture was positive in 3 (7.7%) patients. Among these positive blood cultures, one showed resistance to clindamycin and macrolides, one to nitrofurantoin, and one to cephalosporin and carbapenem. Colistin (also called polymyxin E) was administered in 2 (5.1%) patients, who were positive for *Acinetobacter* in the tracheal discharge and endotracheal tube cultures (Table 1).

Based on the findings, birth weight, gestational age, length of hospital stay, duration of mechanical ventilation, gender, and mode of delivery had no significant relationship with the results of tracheal secretion and endotracheal tube cultures. In a total of 39 neonates, 6 eventually died; none of them had positive tracheal secretion or endotracheal tube cultures. According to the findings, there was no significant relationship between neonatal death and positive tracheal secretion or endotracheal tube culture (*P*= 0.564). The relationship between neonatal death and blood culture showed that only 1 out of 6 neonates who died, had positive blood cultures, and no significant correlation was found between neonatal death and positive blood culture (*P*= 0.403).

According to Table 1, tracheal secretion culture was positive in all neonates with a positive endoendotracheal tube culture; there was a significant difference between the groups (*P*< 0.05). In a total of 3 neonates with positive blood culture, those with Gram-negative bacilli and *Staphylococcus saprophyticus* had a negative tracheal secretion culture. In patients with *Streptococcus* in their blood culture, the tracheal secretion culture was also positive for *Enterobacter*. In other patients with positive tracheal secretion cultures, blood culture was negative. No significant correlation was confirmed between positive tracheal secretion culture and positive blood culture (*P*= 0.37).

Table 2 demonstrates the results of chest radiography. Chest X-ray results were significantly different in patients with different endotracheal tube culture results (*P*= 0.001). There was a significant relationship between positive tracheal tube culture and presence of infiltrates on chest X-ray (*P*= 0.019). Also, a significant difference was reported in the results of chest X-ray among patients with different tracheal secretion cultures (*P*= 0.003). There was a significant relationship between positive tracheal discharge culture and presence of infiltrates on chest X-ray (*P*= 0.019). In addition, a significant difference was found in the results of chest X-ray among patients with different blood cultures (*P*= 0.00). However, the relationship between positive blood culture and presence of infiltrates on chest X-ray was not significant (*P*= 0.113).

**Table 2.** The Chest Radiography Results

**Results**	**Infiltration**	**Infiltration at onset of Mechanical Ventilation**	**New Infiltration 48 hours after Mechanical Ventilation**	**New Infiltration 48 hours after Mechanical Ventilation with the previous infiltration**
**Endotracheal Tube Culture**				
Negative	17	12	1	3
Enterobacter	0	0	1	2
Acinetobacter	0	0	0	2
Pseudomonas	0	0	0	1
**Tracheal Secretion Culture**				
Negative	17	12	1	3
Enterobacter	0	0	1	1
Acinetobacter	0	0	0	2
Pseudomonas	0	0	0	2
***Blood Culture***				
Negative	17	11	0	8
Staphylococcus Saprophyticus	0	0	1	0
Streptococcus	0	1	1	0
Gram-negative bacilli	0	0	0	0

## DISCUSSION

In this study, no organisms were found in the majority of patients *in vitro*. Nonetheless, *Pseudomonas*, *Acinetobacter*, and *Enterobacter* were the most common microorganisms. In a study by Murila et al. (12), *Enterobacter*, *Staphylococcus aureus*, *Escherichia coli*, and *Pseudomonas* were the most common bacteria in the secretion cultures; this finding is in consistence with our study. Moreover, in a study by Afjeh et al. (9), *E. coli*, *Klebsiella*, and *Pseudomonas* were the most common microorganisms. In these studies, the number of positive cultures was higher in all neonates, which reflects the high quality of NICU care at the evaluated hospitals in our study.

In positive cultures of tracheal secretions, one of the patients was resistant to cephalosporins, aminoglycosides, nitrofurantoin, and carbapenem, while others were resistant to cephalosporins, aminoglycosides, nitrofurantoin, carbapenem, and fluoroquinolones. In these recent studies, drug resistance was not investigated, indicating the superiority of the recent survey to previous research.

In terms of positive endotracheal tube culture, the most common microorganisms were *Enterobacter*, *Acinetobacter*, and *Pseudomonas*, respectively. One of the patients was resistant to cephalosporins, aminoglycosides, nitrofurantoin, and carbapenem, while others were resistant to cephalosporins, aminoglycosides, nitrofurantoin, carbapenem, and fluoroquinolones. In this regard, Omid et al. (13) showed that coagulase-positive *Staphylococcus*, coagulase-negative *Staphylococcus*, and *E. coli* were the most common microorganisms; however, this finding is not consistent with our study.

In a study by Shehata et al. (14), the most common microorganisms included *K. pneumoniae*, coagulase-negative staphylococci, *Enterobacter*, *Acinetobacter*, and normal flora; however, this finding is not in line with the present study. According to the antibiogram analysis, all isolated Gram-negative organisms were resistant to ampicillin-sulbactam, cefepime, cefotaxime, ciprofloxacin, and piperacillin; the antibiograms were similar in these studies.

In Table 3, some of the studies in this field have been compared (12–16). Some studies (13, 14) revealed that duration of intubation was directly related to bacterial colonization in the endotracheal tube. In our study, due to the low number of positive cultures, no significant correlation was observed between these variables. Moreover, Mohamed et al. (17) reported that tracheal culture was sensitive in the diagnosis of sepsis; however, it was not confirmed in the recent study.

**Table 3.** Compare the number of similar studies

**Study**	**Type of culture**	**N.P**	**N.P.C**	**V.T**	**Weight**	**M.C.M**	**Antibiogram**
Present study	Tracheal Secretion Endotracheal Tube	39	6	7.86d	23.66.6 g	Enterobacter, Acinetobacter, seudomonas	Resistant to cephalosporins Aminoglycosides, carbapenem nitrofurantoin
Friedland et al. (15)	Endotracheal	29	15	5.4d	-	Staphylococcus Epidermidis, Staphylococcus aureus, Pseudomonas	-
Yew et al. (16)	Endotracheal Tube	25	9	4 ± 1.3d	-	Staphylococcus epidermidis, Klebsiella	-
Shehata et al. (14)	Endotracheal Tube	20	16	3.55d	-	Klebsiella, Coagulase-negative Staphylococcus, Enterobacter, Acinetobacter	Resistant to penicillin and cephalosporins
Murila et al. (12)	Tracheal Secretion	205	38	23.7 ± 11.1d	870 ± 262g	Enterobacter, Staphylococcus Aureus, E-Coli	-
Omid et al. (13)	Endotracheal Tube	100	34	-	-	Positive and negative Coagulase staphylococci, E-Coli	Susceptible to cephalosporins

N.P= Number of Patients N.P.C= number of positive cases V.T= Ventilation Time M.C.M= The most common microorganisms

## CONCLUSION

We concluded that evaluation of tracheal secretion culture is a simple and proper strategy for the diagnosis of VAP. Considering the increased risk of infection in many intubation and extubation procedures, this approach, in addition to reducing the costs, can play a significant role in reducing the incidence of infections.
